# Integrating Domain Knowledge into Deep Learning for Skin Lesion Risk Prioritization to Assist Teledermatology Referral

**DOI:** 10.3390/diagnostics12010036

**Published:** 2021-12-24

**Authors:** Rafaela Carvalho, Ana C. Morgado, Catarina Andrade, Tudor Nedelcu, André Carreiro, Maria João M. Vasconcelos

**Affiliations:** Fraunhofer Portugal AICOS, Rua Alfredo Allen, 4200-135 Porto, Portugal; rafaela.carvalho@fraunhofer.pt (R.C.); ana.morgado@fraunhofer.pt (A.C.M.); catarina.andrade@fraunhofer.pt (C.A.); nedelcu_tudor_ionut@yahoo.com (T.N.); andre.carreiro@fraunhofer.pt (A.C.)

**Keywords:** teledermatology, risk prioritization, skin lesion classification, domain knowledge, hierarchical learning, curriculum learning

## Abstract

Teledermatology has developed rapidly in recent years and is nowadays an essential tool for early diagnosis. In this work, we aim to improve existing Teledermatology processes for skin lesion diagnosis by developing a deep learning approach for risk prioritization with a dataset of retrospective data from referral requests of the Portuguese National Health System. Given the high complexity of this task, we propose a new prioritization pipeline guided and inspired by domain knowledge. We explored automatic lesion segmentation and tested different learning schemes, namely hierarchical classification and curriculum learning approaches, optionally including additional patient metadata. The final priority level prediction can then be obtained by combining predicted diagnosis and a baseline priority level accounting for explicit expert knowledge. In both the differential diagnosis and prioritization branches, lesion segmentation with 30% tolerance for contextual information was shown to improve classification when compared with a flat baseline model trained on original images; furthermore, the addition of patient information was not beneficial for most experiments. Curriculum learning delivered better results than a flat or hierarchical approach. The combination of diagnosis information and a knowledge map, created in collaboration with dermatologists, together with the priority achieved interesting results (best macro F1 of 43.93% for a validated test set), paving the way for new data-centric and knowledge-driven approaches.

## 1. Introduction

Skin cancer incidence has been increasing over recent decades, and according to the World Health Organization, almost three million cases occur globally each year, corresponding to one-third of all diagnosed cancers [1]. The previous facts, associated with the potential risk for misdiagnosis, make the management of skin lesions particularly challenging for both dermatologists and primary care physicians, translating into a considerable economic burden for national health services [2]. In this context, Teledermatology has the potential to improve the efficiency and quality of care at lower costs.

Moreover, major advances in the automatic classification of skin lesions through computer-processed imaging have been recently reported [3,4,5]. Still, most of this work has been conducted primarily at an academic level and mainly focused on specific parts of the problem. One particular topic not yet fully addressed in the literature is the usage of Multimodal Machine Learning approaches [6,7] for dermatological data. Indeed, dermatologists usually make a multimodal decision, as the interpretation of an image is highly influenced by the respective clinical information.

Given this, we can conclude that there is a shortage of systems that convert these different acquired knowledge’s into an effective artificial intelligence-based tool designed to support the referral dermatological process [8]. Furthermore, to achieve a solution with realistic chances of being effectively used in the clinical practice, the design of these systems must take into account the most common practical difficulties during the referral process, such as (1) lack of support during the image acquisition process to ensure image quality; (2) referral decision usually based on incomplete data or not standardized; and (3) inability to manually perform case prioritization in the desired time window, given the demarked discrepancy between the huge number of referral requests and available dermatologists.

The present work integrates a larger project, DermAI, that aims to improve the existing Teledermatology processes between Primary Care Units (PCU) and Dermatology Services in the Portuguese National Health Service (NHS) for skin lesion referral. Through the usage of Artificial Intelligence (AI) and Computer Vision, we envision two major goals: (a) to support doctors in Primary Care Units through the development of a mobile application that fosters image acquisition standardization [9] and (b) to assist dermatologists in the referral process for booking specialist consultations in the hospital through the adequate prioritization of cases. Improving dermatology consultations’ prioritization is particularly relevant in the Portuguese scenario due to the lack of specialists in the NHS and the long waiting lists for this type of consultation.

This research addresses the second goal of the DermAI project regarding case prioritization. Besides its major relevance in the clinical context, the automatic prioritization of dermatological clinical cases is a complex task, usually downstream from the more studied skin lesion classification. To the best of the authors’ knowledge, this work is the first attempt to tackle it with an AI-based solution. In this context, we propose a Deep Learning-based framework to predict the priority level given a clinical image of a skin lesion (with optional patient metadata). The dataset comprises retrospective data from the Portuguese NHS related to referral requests from PCU for the first Dermatology Hospital consultation. Its images are not standardized, and the priority (target) labels add to the complexity since each healthcare site and individual physicians have different requirements for higher priority cases (e.g., available resources). Inspired by how physicians proceed in case prioritization, a skin lesion classification task is included as an intermediate step in the framework. Although extensive work has been carried out in the last decades regarding this goal, it is still an open problem, especially in real-world, uncontrolled data. Accordingly, this study investigates the broader potential of including domain knowledge into the prioritization framework. This knowledge can be used to inspire data-centric preprocessing methods and more robust learning schemes. Moreover, explicit knowledge from clinical experts is used in the form of a novel knowledge map representing the relationship between differential diagnosis and the expected priority distribution, which can then be explored in different ways to guide the final predictions.

The main contributions of this work are summarized as follows:

- an innovative framework for the unexplored and yet-relevant task of dermatological case prioritization;
- a study on how to include implicit and explicit forms of domain knowledge; and
- a novel, explicit knowledge map and its integration to guide model predictions.

## 2. Background and Related Work

### 2.1. Skin Lesion Diagnostic

The skin lesion diagnosis classification paradigm is challenging mainly due to the large amounts of intra-class variability and complex textures and geometric structures. Furthermore, the discrepancy in the number of images of some classes can lead to highly biased Deep Learning models. Over the last few years, there has been an attempt to use transfer learning to mitigate some of the obstacles. The main consensus is that fine-tuning a well-established Convolutional Neural Network (CNN) with high performance in a large dataset, such as ImageNet [10], in a small dataset can lead to a speed-up training and can improve the performance of the models. In Lopez et al. [11], a pre-trained VGG16 was used to perform the binary malignant vs. benign classification. During training, the first four layers of the model were frozen and the rest were trained normally on the ISIC 2016 dataset [12]. This procedure achieved a sensitivity value of 78.66%, which was a much higher value than previously recorded. In Nedelcu et al. [13], the networks were trained by gradually unfreezing the network blocks. Each block was trained for three epochs and then the previous block was unfrozen and trained. This approach avoids destroying any of the information in the pre-trained layers.

Other work used pre-trained networks coupled with linear classifiers [14,15] in order to achieve better results. Menegola et al. [14] applied the VGG-M [16] with a Support Vector Machine (SVM) for the classification of the EDRA dataset [17]. An interesting finding is that using only ImageNet as a pretraining dataset leads to better results than using other medical datasets (Kaggle Retinopathy dataset [18]). Kawahara et al. [15] used a similar strategy by fine-tuning an AlexNet network [19] pre-trained on ImageNet to the Dermofit dataset [20], which resulted in high accuracy in its 10 classes.

The aggregation of information from different types of dermatological images and data modalities has also been attempted, inspired by the fact that, in clinical practice, dermatologists also combine visual inspection (dermoscopic and macroscopic view) with additional clinical information about the patient. Mahbod et al. [21] used two Inception-V3 [22]: one for macroscopic images and the other for dermoscopic images. Nedelcu et al. [23] used this technique coupled with pretraining in ISIC 2019 [24,25,26] to achieve higher results. In addition, Kawahara et al. [5], Kharazmi et al. [27], and Yap et al. [28] employed multimodal learning approaches, integrating patient metadata to enhance performance.

### 2.2. Incorporating Domain Knowledge

Despite the great successes demonstrated by Deep Neural Networks, some of them presented above, it is still challenging, not to say impossible, to guide the learning process towards specific decision paths. Feature engineering is not required for such models and when data are available in sufficient amounts and quality. While this is a clear advantage for the efficiency of the modeling process, we may miss essential inputs that would benefit the final solution. On the other hand, data are often limited and of poor quality, especially in the medical field. In this context, there are interesting attempts at incorporating knowledge from the domain into the learning process, hoping to achieve better suited and robust models.

There are three major approaches to achieving the integration of expert knowledge into Deep Learning processes: relational, logical, and scientific [29]. The first explores relationships between entities (e.g., Melanoma is a malignant skin lesion), defined via relational databases or knowledge graphs. In turn, these can be leveraged through different techniques, such as statistical relational models (Probabilistic Graphical Models—PGMs—or Bayesian Networks) or using learned embeddings, where each entity is represented by a vector in a latent space, learned while accounting for the entities’ context. Interestingly, PGMs and Bayes Nets can also be used to encode Logical Knowledge, besides Probabilistic Context-Free Grammars or Markov Logic Networks. This type of propositional knowledge (e.g., if a skin lesion is asymmetric, showing irregular borders, inconsistent color, etc., the probability of being malignant increases) can be integrated into DNNs, for example, via distillation (student-teacher framework) [29]. Finally, scientific knowledge involves existing models (e.g., Newton’s laws or Navier–Stokes fluid dynamics equations), represented by partial and stochastic differential equations (PDEs), conservation laws and principles, or general invariances. These can be learned from data and used to regularize the learning process of DNNs, via constraints. As an example, Li et al. [30] proposed a domain-knowledge-guided recurrent neural network (DG-RNN) to predict clinical risks based on Electronic Health Records (EHR) and using a medical knowledge graph to improve results and their interpretation by the clinicians.

A survey by Xie et al. [31] summarized different approaches to incorporating domain knowledge into Deep Learning based on medical imaging. Analyzing the different strategies, one can observe that some use external knowledge to inspire the learning process, while others actually use that knowledge to guide the model predictions. Examples of the former include Transfer Learning, where other datasets are leveraged to improve training; Multi-Task Learning, where different goals drive a common underlying sub-model, leveraging different characteristics of the domain (e.g., simultaneous skin lesion classification and segmentation tasks); Curriculum Learning [32,33,34], inspired by existing training patterns of human experts (as discussed in more detail below); and Hierarchical Classification [35,36,37,38,39,40], which explores the fact that some categories under study might have similar clinical manifestations, thus sharing common features. On the other hand, other strategies rely on expert knowledge to improve the network’s results. Different types of domain knowledge can be explored, such as areas of interest in medical images, hand-crafted features known to the experts, or domain priors (e.g., anatomical priors including shape, position, etc.). These can then be explored in different ways, including attention mechanisms, feature fusion techniques, and regularization terms (e.g., constraints). Another recent survey by Dash et al. [41] considers two broad categories regarding the representation of domain knowledge for DNNs: logical constraints and numerical constraints. The former can further be divided into propositional and predicate logic (including binary and n-ary relations). The latter can be subcategorized into loss functions (regularization) and constraints on weights (including priors and transfer learning). Still concerning the integration of domain-based constraints into DNNs, Muralidhar et al. [42] proposed Domain Adapted Neural Networks (DANN), attempting to find a balance between the usual inductive loss and a domain-specific loss. The authors explore monotonic relationships between process variables (monotonic constraints) and information on the normal quantitative range of operation of those variables (approximation constraints). The results showed significant improvements over domain-agnostic networks.

#### 2.2.1. Automatic Lesion Segmentation for Diagnosis

Artifacts such as hairs, veins, ruler markings and air bubbles, or non-target lesions in dermoscopic images can potentially deceive a model’s classifier. Skin lesion segmentation and subsequent selection of an area of interest have been considered to help discriminate between lesions without the interference of such background noises [43]. However, the correct segmentation is not trivial owing to the same challenges. Fully CNNs (FCNs) have been the staple method used in the skin lesion segmentation challenge. Most methods are based in the U-Net [44] with small modifications [45,46]. In Öztürk et al. [47], a FCN used spacial information to segment images in full resolution and without any pre- or post-processing. In Zahra et al. [48], an ensemble of Bayesian FCN was used to take advantage of the annotator-related biases to achieve a gold standard in image segmentation. Several ground truths, for the same image, were used to train several networks, which were then fused to obtain the final prediction. Other work also utilized the fusion of several image scales to improve the quality of the segmentation [49]. Lastly, the difference between image acquisition formats was also analyzed. In Andrade et al. [50], a model for the precise segmentation of lesions in macroscopic skin lesion images was developed, tackling the problem of the limited amount of mobile acquired/macroscopic images by making use of the sizable number of dermoscopic images. For this, a Cycle-Consistent Generative Adversarial Network (CycleGAN) [51] was used for the translation between the two types of dermatological images: macroscopic and dermoscopic. The results of this technique established a new state-of-the-art performance in the SMARTSKINS dataset [52].

Subsequently, the correct selection of an area of interest for use during the diagnostic classification after the segmentation was also analyzed. Yu et al. [53] proposed a two-stage framework, integrating very deep lesion segmentation and classification networks. The first allowed them to predict the lesion masks, to crop the dermoscopic images into square and tight lesion patches, and to send them as input for the classification stage to extract more representative and specific features within the lesion regions. The experimental results reported that the segmentation task led to significant performance improvement in lesion classification. Burdick et al. [54] also investigated the role of segmentation in CNN-based lesion classification, specifically the effect of expanding the segmentation border to include neighboring pixels. The results suggest that such pixels provide contextually relevant information and that border enlargement is beneficial to a certain extent, with dilated masks achieving higher performance than dermatologist-like segmentation masks. In the work of Tang et al. [55], a Global-Part Convolutional Neural Network (GP-CNN) model was developed. The motivation behind this design was to treat local information and global context information with equal importance. The Global-CNN was trained on resized skin lesion images, with its results extracting global context and producing Class Activation Maps (CAMs). A CAM-guided probabilistic multi-scale cropping method was employed on the original dermoscopic images to obtain skin lesion patches, which were used to train the second sub-model, Part-CNN, and to capture local-scale information of skin lesion regions. The results from the two sub-models were finally fused by the weighted ensemble strategy, enhancing classification performance.

#### 2.2.2. Hierarchical Classification

Traditional CNN-based classification systems assume that all classes are equally hard to distinguish, compromising the classification of visually similar categories. Therefore, to enhance CNN’s performance, hierarchical systems have been considered in recent years, having already proved their effectiveness over flat classification approaches. These systems decompose the global classification problem into multiple sub-problems in a hierarchical manner, aiming to preserve some relationships that exist across various categories. Yan et al. [35] introduced Hierarchical Deep Convolutional Neural Networks (HD-CNN), which incorporate deep CNNs into a hierarchy of categories by using coarse and fine category classifiers to distinguish easy and difficult classes, respectively. In the work of Zhu and Bain [36], a system called Branch Convolutional Neural Network (B-CNN) was proposed. This system introduces branches along the main CNN to make multiple predictions hierarchically. This strategy was applied by Sali et al. [37] to diagnose gastrointestinal disorders on histopathological images. In the medical field, An et al. [38] developed a training framework for disease detection that employs transfer learning between models from different hierarchical levels. With respect to skin lesion diagnosis, Fisher et al. [39] proposed a method that uses a hierarchical decision tree with a K-NN classifier at each decision node to categorize different types of lesions. Furthermore, Barata et al. [40] proposed a hierarchical methodology for skin cancer diagnosis, in which two different hierarchies were explored. The benefits of using a structured classification over a multi-class problem were demonstrated, making it possible to achieve higher results.

#### 2.2.3. Curriculum Learning

As previously mentioned, another approach that intends to mitigate the differences verified in the classes’ classification difficulty consists of mimicking the human gradual learning process, starting from the easiest tasks and gradually progressing to the hardest ones. This learning procedure is called curriculum learning [32] and has been recently considered in the medical image field. Maicas et al. [33] proposed a teacher–student curriculum learning system for automatic breast screening classification, which trains on simpler tasks before introducing the malignancy detection problem. The work developed by Tang et al. [34] employed an attention-guided curriculum learning framework that builds the curriculum based on the disease severity level extracted from radiology reports. Curriculum learning was also used by Tang et al. [56] for skin lesion segmentation, aiming to handle the overfitting problem caused by the imbalanced number of difficult and easy samples.

## 3. Materials and Methods

**Figure 1:**
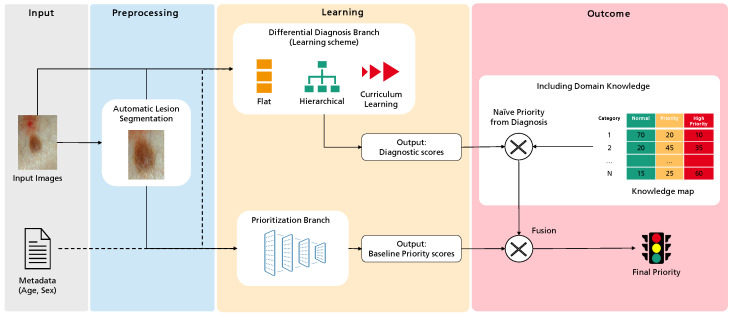
Overview of the methodology followed in this work towards the prediction of dermatological case risk prioritization.

Recalling the main objective of this project—improving case referral from general practitioners to dermatology specialists—the primary goal of this work is to create a predictive model to classify the priority of a case into one of three levels: normal, priority, or high priority. Figure 1 illustrates the overall workflow of this process, highlighting four major sequential blocks: Input, Preprocessing, Learning, and Outcome. The first concerns the input data, the case properties explored for the intended task. This work considers a dermatological image and associated metadata such as the patient’s age and sex. Then, these data are subject to several preprocessing operations. These can be standard to Deep Learning, such as normalization, or specific to this work, as the optional automatic lesion segmentation and data augmentation. Then, the Learning block comprises the two branches where actual learning takes place: the differential diagnosis branch, where different learning schemes are explored, and the output are an array of scores for the set of categories under analysis, and the priority branch, which can be seen as the baseline for the target task, connecting the inputs to the priority scores output. Finally, we propose an additional Outcome block, where the different branches can be combined with domain knowledge to achieve the final priority level prediction. In this case, domain knowledge is based on a statistical distribution mapping each differential diagnosis to priority priors. Details on this map are provided below.

### 3.1. Database

**Figure 2:**
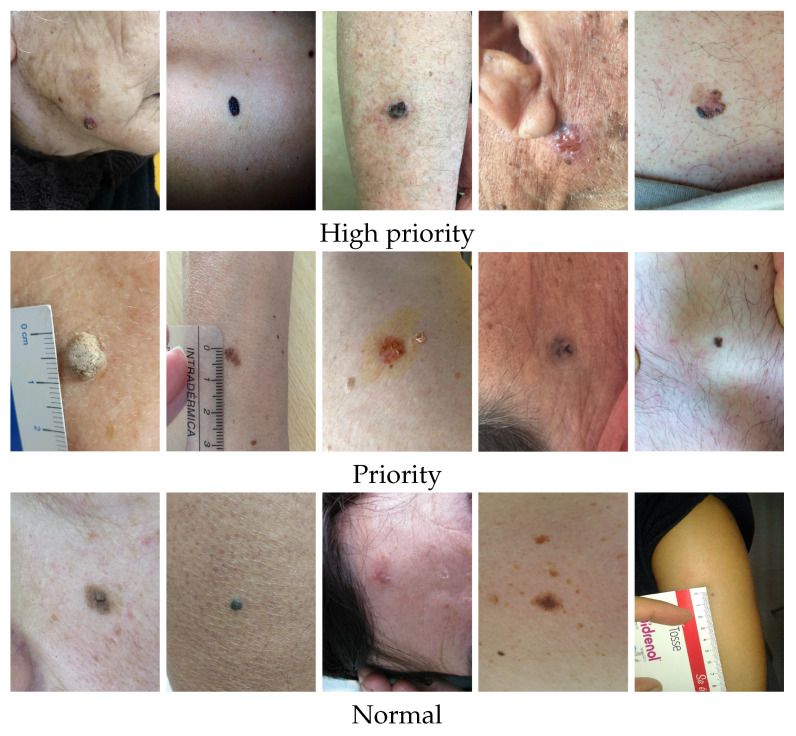
Illustrative examples of lesions from the DermAI dataset with different priority levels.

The authors had access to anonymized retrospective data from the Portuguese National Health System related to the referral requests from Local Health Care Units for the first Dermatology Hospital consultation. The referral requests that occurred between 2014 to 2020 (before COVID-19 pandemic) and only cases corresponding to single lesions with differential diagnosis available were considered. The authors counted with the support of a group of dermatologists to select the clinically relevant list of differential diagnoses to be considered when building a prioritization model for dermatological referrals. The DermAI dataset used here was first presented in [13] and consisted of a total of 3427 cases corresponding to single lesions from 13 distinct differential diagnoses. The average age (and standard deviation) of the individuals correspond to 55.84 ± 22.18, and regarding the sex, there are 1406 male and 2021 female cases. The dataset contains mostly macroscopic (close-up) images of lesions; however, in some cases, the images are anatomical (296). The distribution of cases in relation to the differential diagnosis and priority level provided by dermatologists is presented in Table 1, and some illustrative examples of lesions with different priority levels are shown in Figure 2. The differential diagnosis refers to the diagnosis provided by dermatologists after the specialist consultation and priority refers to the level of priority in the triage process to book the consultation. It is important to stress that, although there are national guidelines to define the priority (normal, priority, and high priority), the different hospitals have a different number of specialists in this field and different resources so these data include this intrinsic variability.

Observing the partition distributions in Table 1, it is clear that the sample distribution per differential diagnosis and priority levels are not balanced. The training set and test set 1 were stratified so that their distributions matched. However, due to the low representation of some diagnostic classes and priority levels, we decided to select a subset of the test set 1 to balance the priority levels. Furthermore, due to the mentioned challenges of the priority labels, we asked three experienced dermatologists to validate 200 of these samples regarding expected priority. Challenging examples where there was disagreement were not considered in this subset. This resulted in test set 2, comprised of 192 samples, distributed among high priority (all 41 samples available from the test set 1), priority (all 70 samples from test set 1), and normal (81 samples randomly sampled from the test set 1). Additionally, due to some diagnostic categories being greatly underrepresented, we found that the creation of a validation set made the training even more challenging (Solar Lentigo has only 39 training samples, and Melanoma has 44, for example) or left the final test set too small (9 testing samples for Solar Lentigo, and 8 for Melanoma). Thus, when comparing the impact of different approaches and techniques, we rely on the results of test set 1 (more general, unbalanced, and many labels unvalidated) and report the final performances on test set 2 (fewer samples, more balanced, and validated labels).

Finally, bearing in mind that the priority level in different hospitals can change depending on the available resources, the authors worked closely with a group of three experienced dermatologists in order to build a knowledge map of the priority level for each differential diagnosis. We asked the dermatologists to individually provide their priority level distribution depending on the differential diagnosis. In Table 2, we present the mean and standard deviation of the obtained results. Looking at the Seborrheic Keratosis class (1 SebKer), the doctors agree that 80% of the cases fall into the normal class while 17% are noted as priority and a minority, 3%, are considered high priority. Oppositely, we can see that Malignant Melanoma class (13 MM) was mostly referred to as high priority, with a few (10%) being referred as priority. Moreover, observing standard deviations of these two classes, it is possible to see that the three doctors mostly agree on this distribution. There are some classes though, such as Neoplasm of Unclear Behavior (6 UncNeop) or Other Malignant Neoplasms (11 OtMalNeop), where the distribution is more evenly spanned across two priority levels: normal and priority for the former and priority and high priority for the latter.

### 3.2. Automatic Lesion Segmentation for Diagnosis Enhancement

Preprocessing transformations such as lesion segmentation may enhance the performance of CNN models, as proven by the positive results of the works presented in Section 2.2.1. Furthermore, given that this is still an open issue in image analysis, we investigated the application of a segmentation step followed by a crop around the lesion in the images of the dataset to verify whether the removal of those structures is advantageous for differential diagnosis.

In order to obtain segmentation masks for the DermAI dataset, a procedure based on two methods was implemented. First, an attention-guided methodology using the Gradient-weighted Class Activation Mapping (GradCAM) technique [57] was applied to the flat baseline diagnosis model trained on the original images of the dataset, which is described in the following section. The resulting activation maps were used to extract masks from the classifier, by performing empiric thresholding operations dependent on the average intensity of the grayscale maps. To avoid noise commonly found in the corners of the maps, patches equal to the average intensity of the image were applied on such regions. As lesions are usually located in the center of the image, the closest blob to this point was selected and the GradCAM mask was obtained. For the second approach, we employed a DeepLabV3+ [58]-based segmentation model, with MobileNetV2 [59] as a feature extractor, from Andrade et al. [50], to obtain our DeepLab masks.

The overlap between the segmentation masks obtained from both methods was found. In cases where the overlapping area was above 50% of the GradCAM mask’s area, this mask was accepted; if this condition was not verified but the overlapping area was more than 60% of the DeepLab mask’s area, then the latter was taken. Otherwise, an empty mask was considered, which means that the original image was used.

**Figure 3:**
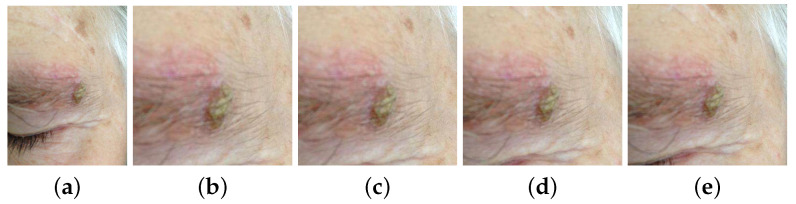
Examples of original and cropped images with different tolerances. (**a**) Original image. (**b**) Cropped with no tolerance. (**c**) Cropped with 10% tolerance. (**d**) Cropped with 30% tolerance. (**e**) Cropped with 50% tolerance.

The location of the lesion was inferred from the mask. A square image patch containing the whole skin lesion was defined by finding the difference between the largest and smallest dimensions of the bounding box and by adding half of this value to both sides of the latter, except in cases where this was not possible due to the total dimensions of the image. Furthermore, a tolerance based on a percentage of the approximate radius of the lesion was studied, as neighboring pixels may include contextually relevant information. Each square area of interest was used to crop the original images for both training and testing of the differential diagnosis models explained in Section 3.3.1. The lesion patches experimented on are presented in Figure 3: original image; cropped image with no tolerance; and cropped with the inclusion of neighboring pixels corresponding to 10%, 30%, and 50% of the lesion radius.

### 3.3. Differential Diagnosis Branch

#### 3.3.1. Flat Approach

The EfficientNet is a group of networks developed based on the network scaling (depths, width, and resolution), achieving state-of-the-art results while being multiple times smaller and faster [60]. We chose to employ EfficientNetB3 as it requires a much lower number of parameters to achieve the performance of other commonly adopted CNN architectures on the ImageNet dataset.

To accommodate an EfficientNetB3 network for skin lesion diagnosis, a fully connected layer was applied on top of the extracted feature map, generating a number of channels related to the number of classes to predict (13). For dimensionality reduction, the Global Average Pooling method was applied, as it is known to reduce overfitting [61]. The final output was obtained by using the softmax activation function.

The role of a lesion segmentation step in skin lesion diagnosis was investigated by training models with each of the following inputs: original images of the DermAI dataset and cropped images with 0%, 10%, 30%, and 50% tolerance (Figure 3). The input of the EfficientNetB3 architecture consists of images of size (300 × 300); hence, the considered images are resized to the desired shape using the nearest neighbor method. To mitigate overfitting issues due to the imbalance of the dataset, we employed stratified batches, i.e., each batch always holds exactly one sample of each skin lesion class, resulting in a batch size that matches the number of classes for the dataset (13). We decided to use 200 samples per class as it was a reasonable compromise between oversampling of the classes with fewer examples and downsampling of the opposite. Additionally, simple data augmentation transformations were applied: rotation in the range of [1, 30] degrees, horizontal and vertical flip, shear distortion, zooming, width and height shift in the range [0, 0.2], channel shift in the range [0, 10], brightness in the range [0.2, 0.8], and reflect as the fill mode.

The network was trained using the weights pre-trained on ImageNet. The frozen block approach was adopted for better results [62]: each EfficientNetB3 block was trained for three epochs using a learning rate of $10^{-4}$ for the top layers and $10^{-5}$ for the rest of the blocks. Training stopped when validation loss no longer yielded an improvement and the model started overfitting. Adam was used as the optimizer, and categorical cross-entropy was specified as the loss function.

The input images resulting in the best performing flat model were adopted for the learning schemes described below. As further discussed later, the choice of these learning approaches was inspired by clinical and intuitive procedures, intending for the introduction of domain knowledge to improve the final priority assessment.

#### 3.3.2. Hierarchical Classification

**Figure 4:**
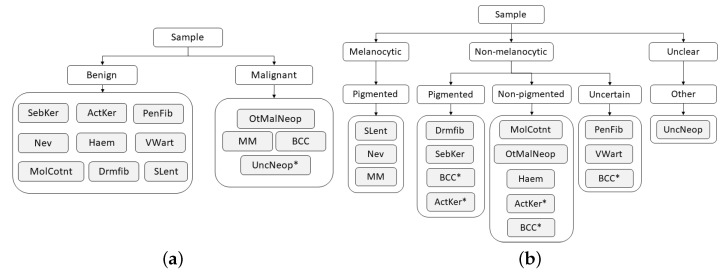
Hierarchies considered for skin lesion diagnosis. (**a**) Two-level hierarchy. (**b**) Three-level hierarchy.

To address the challenge of some types of skin lesions having similar clinical manifestations, dermatologists usually consider the intrinsic hierarchical organization of these lesions when performing their diagnosis. Therefore, to mimic the medical procedure, the dataset was hierarchically organized according to some of the methodologies used by clinicians, with the aim to facilitate the classification of the lesions. This organization was made in collaboration with the three experienced dermatologists, resulting in a two-level and a three-level hierarchy, as illustrated in Figure 4.

Although the process of dividing lesions into different coarse categories can make it easier for dermatologists to diagnose skin lesions, due to their complexity, this is not a straightforward process, and for this reason, some exceptions may arise (marked with * in Figure 4). With respect to the two-level hierarchy (Figure 4a), as its name suggests, the Neoplasm Unclear Behavior class was assigned to the malignant set by the fact that its categorization is not clear. Concerning the three-level hierarchy (Figure 4b), despite the majority of Basal Cell Carcinoma lesions being non-pigmented, there may also be cases in which these lesions are pigmented or even uncertain. The same occurs in Actinic Keratosis lesions, which may present both pigmented and non-pigmented characteristics. Therefore, for these two classes, each example was carefully analyzed by a dermatologist who assigned it the correct coarse label (pigmented, non-pigmented, or uncertain).

After the dataset was organized, the B-CNN approach [36], introduced in the related work, was employed, as it already proved to be effective for medical image classification [37]. In this strategy, new branches were inserted along the main network to predict as many labels as the number of hierarchical levels. Assuming that low-level features are captured by shallow layers and high-level features are captured by deeper layers, the predictions are ordered from coarse to fine categories. As in the flat classification approach (Section 3.3.1), the EfficientNetB3 network pre-trained on the ImageNet was employed as the main network. Regarding the two-level hierarchy (Figure 4a), a new branch was introduced between the fifth and sixth blocks of the main network to predict between benign and malignant lesions, and, in the case of the three-level hierarchy, two new branches were introduced to the main network to predict the coarse categories relative to the upper hierarchical levels. In this case, one of the new branches was connected between the fourth and fifth blocks of the network to predict the first-level categories (melanocytic, non-melanocytic, and uncertain), and the other one was between the fifth and sixth blocks to predict the categories corresponding to the second hierarchical level (pigmented melanocytic and non-melanocytic lesions, non-pigmented, uncertain, and other). Similar to the original implementation [36], for both hierarchies these branches were composed of three fully connected layers, having dropout and batch normalization techniques also applied to prevent overfitting and to stabilize the learning process. For both hierarchies, fine categories, i.e., the lesion diagnosis, were predicted on the top of the EfficientNetB3. As a training protocol, the frozen block approach introduced in Section 3.3.1 was also adopted in this part of the work, and the same learning rates and optimizer were employed. In this case, the blocks were unfrozen up to the group before the introduction of the first branch. As loss functions, the categorical cross-entropy was considered. The final loss consisted of a weighted summation of all prediction losses (coarse and fine), so throughout the learning process, different loss weights were assigned to each level of the hierarchy to determine the corresponding contribution to the final loss. The change in weights was made to optimize the learning of the coarse categories in the initial epochs and, as the learning process evolves, to shift the optimization focus to the fine categories. Hence, in the case of the two-level hierarchy, the loss weights started out as [0.98; 0.02]; after six epochs changed to [0.3; 0.7], the 12th was set to [0.1; 0.9] and, finally, the 18th epoch changed to [0.0; 1.0]. Concerning the three-level hierarchy, the loss weights began as [0.97; 0.02; 0.01], in the 6th epoch, changed to [0.1; 0.8; 0.1]; in the 12th epoch, changed to [0.1; 0.2; 0.7]; and from the 18th epoch until the end of the training process, followed [0.0, 0.0, 1.0].

Moreover, the strategy proposed in [38] was also investigated. However, as the results fell short of expectations, we decided not to include them in this paper.

#### 3.3.3. Curriculum Learning

The visual similarities that exist between different types of skin lesion and the different clinical manifestations that the same lesion may present make some categories more difficult to classify than others. Thus, the use of curriculum learning was also explored in this work, intending that categories could be learned according to the corresponding difficulty, favoring the learning of harder categories. With the exception of the SLent class, which was considered the hardest category since it is the least represented in the train dataset, the criteria used to define the categories’ difficulty were based on the F1-score achieved with the flat approach (Table 4). Therefore, the learning started with the easiest classes (i.e., the ones that resulted in a higher F1-score, as SebKer, ActKer, and MolCont), and throughout the process, the hardest classes were introduced (being OtMalNeop, BCC, and SLent the last classes introduced).

Similar to the previously described approaches (Section 3.3.1 and Section 3.3.2), the EfficientNetB3 network with ImageNet weights was first employed and the training procedure was also based on the frozen block procedure, which gradually unfreezes groups of the network’s blocks. Each block was trained for three epochs, and in this case, a total of six blocks were unfrozen. The learning process started with four classes, and after all of the desired groups had been unfrozen, a new class was introduced in the dataset, according to the established ordering. When introducing a new class, all blocks were refrozen and the aforementioned unfreezing process was repeated. As training protocol, the Adam optimizer was considered and the categorical cross-entropy loss function was used. Regarding the learning rate, it was tuned to 1 × 10${}^{-4}$ on the top layers and 1 × 10${}^{-5}$ for the rest of the blocks. With respect to the batch size, a batch size of 13 was considered, ensuring that, after all classes were introduced, one sample from each class was presented at each batch of images.

#### 3.3.4. Multimodal Learning

The previously described strategies can be further developed to incorporate clinical information as additional input, since the combination of complementary information from different data modalities has been shown to slightly improve performance [23,27,28]. Our networks are set to receive two inputs: dermatological images and patient-level metadata (age and sex). To merge this information, we employ a late feature fusion technique [63]. The patient metadata is directly concatenated with the feature vector extracted from the dermatological images by the EfficientNetB3 architecture; subsequently, the merged information is provided to the classifier. The aforementioned training protocols for each learning scheme (Flat—Section 3.3.1, B-CNN—Section 3.3.2, and Curriculum—Section 3.3.3) are employed.

### 3.4. Prioritization Branch

The prioritization branch is an end-to-end approach to ascertain the priority level of a skin lesion, without considering its diagnostic. Therefore, the EfficientNetB3 network, with weights trained on ImageNet, is trained with the priority labels described in the last line of Table 1. The pre-trained model was modified to comply with the three desired classes (normal, priority, and high priority), and a softmax activation function was used in the output. Similar to the diagnostic approaches, the frozen block approach was used. Initially, only the last three blocks of the EfficientNetB3 were trained for three epochs, and gradually more groups were unfrozen for six cycles, meaning that, in total, six blocks were trained. Additionally, the Adam optimizer in combination with categorical cross-entropy loss was used with a small learning rate to prevent the rapid overfitting in the first blocks. The stratification of the imbalanced dataset was made in consideration of the priority and a batch size of 12 was used, ensuring that each priority class has four samples per batch.

Last, the role of lesion segmentation and the incorporation of clinical information in the prioritization branch was also tested. For the former, two types of cropping were considered: the original images and the best tolerance of cropping identified on the Flat differential diagnostic approach.

### 3.5. Final Priority (Integration with Domain Knowledge)

The incorporation of explicit domain knowledge in the final priority prediction given by the proposed framework can be performed in a number of ways. The naive method of including information from the experts is to combine the results of the differential diagnostic class and the knowledge map shown in Table 2. Since this knowledge map represents the average priority given to a skin lesion depending on its class, this approach is similar to the routine of an expert dermatologist when analyzing skin lesions. Initially, a diagnosis is ascertained, and depending on it, a priority is given. In the naive approach, the diagnostic scores are multiplied by the distribution average scores, ending with an aggregated priority level prediction. Here, each class score, which results from the learning block of the differential diagnostic branch, is multiplied by the corresponding knowledge map average values for the three priority levels. Then, the final priority prediction is computed by aggregating these results, through a summing operation or by taking the max value for each priority level.

Nonetheless, the naive approach does not consider any learning regarding the prioritization task, as it only considers the learned diagnostic branch and the knowledge map. A simple way to include the baseline priority is to fuse the outputs of the baseline priority model and the naive approach. To achieve this, a weighted addition on the outputs was performed, as in αPriority+βDiagnosis. Therefore, this approach, entitled Simple Approach, utilizes two models: one for prioritization and one for diagnosis.

The last approach tested (Combined Approach) includes the learning step during the training of the prioritization task. For this, an auxiliary branch can be added to the differential diagnosis model to compute the prioritization. This ensures that the model learns the intricate relationship between the class and the priority of the lesions. Thus, the Combined Approach has two branches: the first computes the diagnosis and the second computes the prioritization. These branches are then joined to learn the final priority. The diagnostic output is combined with the knowledge map and added, with the expression used in the previous approach, to the prioritization branch output. This addition is made during training, and the loss function takes into consideration both diagnostic and prioritization, making sure that the diagnosis is not prejudiced for the priority.

## 4. Results and Discussion

### 4.1. Differential Diagnosis Branch

#### 4.1.1. Preprocessing Strategies

The overall metrics score results for the different experiments on skin lesion segmentation for diagnosis improvement are summarized in Table 3. The flat approach based on EfficientNetB3, pre-trained on ImageNet, was trained with the original images of the dataset and returned accuracy of 37.59%, weighted F1 of 40.49%, and macro F1 of 26.11%. Considering the model trained on square lesion patches with 0% tolerance, accuracy and weighted F1-scores dropped from 37.59% to 34.80% and 40.49% to 37.63%, respectively, whereas macro F1 registered a slight improvement. The introduction of 10% tolerance further decreased the performance, with this model showing the worst overall results (absolute difference of, at best, 1% in all considered metrics). The ‘dilation’ of the area of interest to include more context proved to be beneficial when considering higher tolerance values (30% and 50%). Both accuracy and weighted F1 achieved the best overall values for the first; however, there is a 1% absolute difference in macro F1 with an advantage for the 50% tolerance images. Interestingly, the usage of zero or small (10% tolerance) resulted in worse classification performance but achieved better results when higher values of tolerance were used, which suggests that a specific amount of contextual information is advantageous for the model. As an example, the computation of specific features related to color and texture variations requires pixel information of both lesion and skin.

Analyzing sensitivity, precision, and F1 per class for the models with best overall results (trained on 30% and 50% tolerance images), the first presented the best scores for the majority of the malignant classes (OtMalNeop and MM), thus being considered the top-performing approach. The metrics for the best model (cropped images with 30%) are illustrated in Table 4. Despite the data-balancing techniques, there is a considerable imbalance between the different classes’ scores. Regarding Seborrheic Keratosis (SebKer), which is the most represented class in the dataset, it obtains the highest F1-score (53.23%) with some misclassifications in Nevus (Nev), Dermatofibroma (Drmfib), Neoplasm of Uncertain Behaviour (UncNeop), and Malignant Melanoma (MM). Actinic Keratosis (ActKer) achieves the second-best F1-score (52.57%), and typical misclassifications include Basal Cell Carcinoma (BCC), Other Malignant Neoplasms (OtMalNeop), and SebKer. The Viral Warts class (VWart) is one of the best classified categories (F1 44.90%), with some examples being misclassified as UncNeop. Nevus class, the second most represented in training, achieved an F1-score of 39.82% with some misclassifications to SebKer, Drmfib, and VWart. Additionally, Dermatofibroma achieved an interesting performance of 36.00% for F1. Although with almost 100 examples in the training set, Pendulum Fibroma (PendFib) only achieved a F1-score of 27.10%, with misclassifications spanning across several benign classes. From the benign classes, the ones with fewer examples in the dataset are Molluscum Contagiosum (MolCont), Haemangioma (Haem), and Solar Lentigo (SLent) achieving poorer performances (22.73%, 14.29%, and 8.7%, respectively). Regarding Neoplasm of Uncertain Behaviour (UncNeop), which includes higher intra-class variability, a lower F1-score (16.84%) was obtained. The malignant class of Other Malignant Neoplasm (OtMalNeop) also achieved a low F1-score (10.34%), with most misclassifications falling into SebKer, ActKer, Drmfib, and BCC. The BCC class achieved the lowest F1-score, 3.85%, which can be explained by the small number of samples (less than 50) and higher variability in their biological and clinical manifestations. Finally, MM achieved an interesting F1-score of 26.32%, despite the low number of training examples and common misclassifications that fell into Nev and SebKer, which is in accordance with the clinical point-of-view. These results are in line with the results previously presented in the study of Nedelcu et al. [13].

#### 4.1.2. Comparison of Learning Schemes

Taking into account the previous results achieved with the flat classification approach, the cropped images with 30% tolerance were adopted as input images both in the case of the hierarchical classification and curriculum learning strategies. In Table 5, the overall scores achieved with the different experiments for skin lesion differential diagnosis may be found.

Analyzing the results concerning the hierarchical classification (B-CNN), it is demonstrated that the results of the flat classification approach, which was used as the baseline, were outperformed with both hierarchies (two and three levels). In the case of the three-level hierarchy, an absolute difference of almost 3% is verified in terms of accuracy, 2% in weighted F1, and with respect to macro F1, the improvement corresponded to around 2.5%. Moreover, comparing the two hierarchies, although the three-level hierarchy achieved slightly higher results in terms of accuracy and weighted F1, this difference was about 1.5% at most, demonstrating a similar performance between them.

With respect to the curriculum learning strategy, the gradual learning of the different classes benefited the differential diagnosis, achieving a considerable improvement in the overall metrics, when compared with both flat and hierarchical strategies. Taking the flat classification as the baseline, an absolute difference of 10% was verified in accuracy, 8% in weighted F1, and regarding macro F1, a difference of 6% was observed.

**Figure 5:**
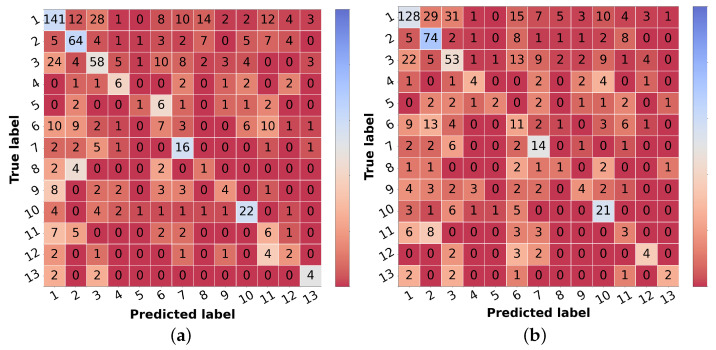
Confusion matrices of test set 1 for the differential diagnosis model trained with curriculum learning. (**a**) Input: image. (**b**) Input: image and metadata.

The corresponding scores for the best learning scheme (curriculum learning) for each diagnostic class are represented in Table 6 in the leftmost columns for each metric (“img”). The confusion matrix may also be found in Figure 5a. Comparing these results with the ones achieved with the flat classification approach (Table 4), it is possible to observe an improvement for most of the classes in terms of precision and F1-score. Regarding malignant lesions, such as OtMalNeop, BCC or MM, this enhancement in F1-score reached an absolute difference of about 8%, 12%, and 14%, respectively. Furthermore, the sensitivity of OtMalNeop or BCC registered an improvement of around 50%.

It is also worth mentioning that, although the structure of the DermAI dataset used in our previous work is not exactly the same as the one considered in this study, as here the data were stratified considering the priority and differential diagnosis, the curriculum learning experiment allowed us to improve the results obtained before [13]. In terms of accuracy and weighted F1, this improvement corresponded to an absolute difference of around 6%, and with respect to the macro F1 score, an improvement of 5% was verified.

Therefore, considering the results achieved with the various experiments, the curriculum learning approach was the learning scheme that demonstrated better performance for skin lesion differential diagnosis.

#### 4.1.3. Multimodal Learning

Confronting the experimental results presented in Table 7 that used image and metadata with the ones achieved with only images (Table 5), it is shown that the addition of patient metadata (age and sex) slightly decreased the overall metrics for skin lesion diagnosis, except for the B-CNN model using a three-level hierarchy. Moreover, although the results did not improve with the introduction of metadata, the curriculum learning experiment still surpassed all other strategies. In Table 6, the impact of metadata on the classification of each of the classes may be analyzed, and in Figure 5b, the corresponding confusion matrix is represented. It is observed that the sensitivity and F1-score were penalized for most of the classes, demonstrating the disadvantage of including metadata for differential diagnosis. With respect to some malignant classes, such as OtMalNeop or MM, drops of 13% and 25% were verified in terms of sensitivity, and in terms of F1-score, this decrease corresponded to around 6% and 9%, respectively. Although BCC class achieved better results using metadata it should be noted that this corresponds to having 2 more correctly classified instances out of 11. Additionally, regarding UncNeop class, although it gave better overall results using metadata, looking at the confusion matrix, we can observe that misclassifications using only images occur more to malignant classes.

Considering those results, the curriculum learning strategy without metadata (using only images) was the learning scheme adopted for differential diagnosis classification in the following experiments.

Nevertheless, as dermatologists also take clinical information into account to perform skin lesion diagnosis, these were not the expected results. We expected that the introduction of metadata could improve the outcomes as previously stated in [5,27,28]. It is, however, important to note that, in these studies, other clinical information such as lesion location, lesion elevation, or lesion size were also considered, which leads us to believe that age and gender may not be sufficient to improve the differential diagnosis in the context of this work.

### 4.2. Skin Lesion Diagnostic—Results in the Literature

As the dataset employed in this work is private, the proposed skin lesion diagnostic model is not directly comparable with other studies in the literature, except for the work of Nedelcu et al. [13], which also reports results on this dataset. Therefore, due to the different datasets used and the variety of skin lesion diagnosis classes, the results’ analysis is challenging. Nonetheless, in Table 8, we summarized the performance of a number of relevant studies conducted in the area, in addition to the work on the DermAI dataset.

Regarding the organization of the diagnostic classes, there is the differentiation between benign vs. basal cell carcinoma vs. melanoma [14], seborrheic keratosis vs. nevi vs. melanoma [40] or even more detailed types of skin lesions, which varied depending on the study [4,5,13,23,28,39]. Concerning the representativeness of the considered classes, several examples from each lesion category were available in some of these work [14,28,40], in contrast with the studies conducted on the DermAI dataset, in which some lesions comprised less than 50 training examples [13].

Moreover, as the employed datasets were not always the same, the images’ difficulty could also differ in the various works, which may have had an influence on the algorithms’ outcomes. For instance, images from the Dermofit database were collected under standardized conditions, whereas images from the DermAI dataset were acquired under different lighting and capture conditions, which increased their variability. Although Esteva et al. [4] also used images from different sources, the training process was made with 757 finer diseases, which demonstrated better performance than a model trained directly on the nine final classes.

Furthermore, concerning the modalities of the images explored in the various studies, some works only considered dermoscopic images [14,39], which, in comparison with macroscopic or anatomic images (DermAI dataset), present less variability caused by features such as facial or anatomic structures. Additionally, in the work of Yap et al. [28], macroscopic and dermoscopic images were considered for each skin lesion, thus providing complementary information. Moreover, in that work, samples with anatomical features (e.g., eyes, multiple facial landmarks, etc.) were removed in an attempt to avoid biases.

It is, then, possible to infer the complexity of the DermAI dataset, which comprises few examples relating to some skin lesion categories, and high image variability caused not only by the various image acquisition conditions but also from the different explored modalities. Nonetheless, as previously mentioned, compared with the other study conducted on the DermAI dataset [13], the model proposed in this work surpassed its performance with respect to all of the considered metrics.

### 4.3. Priority Branch

The overall results from the trained prioritization models are summarized in Table 9. By observing the overall results, one can conclude that the incorporation of metadata proved to be quite disadvantageous. When considering metadata, the models reach under-performing results, leading to a drop of at least 10% in all metrics. Regarding the type of input, an overall conclusion can be drawn from the robustness of the cropping method. Using cropped images with 30% of tolerance seems to improve the classification capacity of the prioritization models, which lead to the top-performing results for each of the simple and metadata model. These results are in line with the results obtained considering the flat approach for differential diagnosis (Section 4.1.1).

For the top-performing model, the metrics and confusion matrix for each prioritization class, shown in Table 10, were analyzed. The main objective in the prioritization model was to have high sensitivity in the priority classes and high F1 in the normal class. This is crucial since a high sensitivity in priority classes means that the high-priority lesions were correctly identified as needing urgent care. Likewise, the high precision in the normal class means that the cases are not classified as more urgent than needed. With respect to the high priority class, it is possible to ascertain that more than half of the cases were correctly identified. However, in the confusion matrix, it is possible to observe that most incorrectly classified cases were given a normal classification, which is the least desired outcome. Moreover, in the normal class, there are still a high number of misclassified cases.

These results are a product of the intrinsic variability of the gathered data, meaning that, depending on the hospital resources (facility and experts availability), the priority level attributed might change.

### 4.4. Final Priority

**Figure 6:**
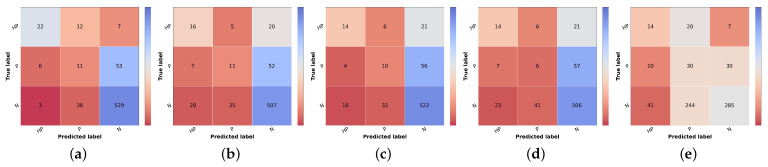
Confusion matrices of test set 1 for the different risk prioritization approaches tested. (**a**) Naive GT. (**b**) Naive (Max). (**c**) Naive (Sum). (**d**) Simple Approach. (**e**) Combined Approach.

The results from the combination of the trained models with the knowledge map to obtain the final prioritization are summarized in Table 11, and the results per class are summarized in Table 12. The corresponding confusion matrices can be found in Figure 6. In these tables, the first line represents the result obtained in the prioritization branch (baseline priority) of which the results were discussed in the previous section.

The result of the ground truth of the diagnostic and the knowledge map (KM) (KM presented in Table 2) is also shown, named Naive GT, since it represents an upper bound of the diagnosis branch, meaning that it is the maximum value of prioritization possible to obtain considering the real values of diagnosis (ground truth). Concerning the naive approach, the model trained with the curriculum learning strategy without metadata (Table 5) was used to compute the differential diagnosis and then combined with the knowledge map to compute the prioritization. As stated in the methodology, two types of combinations were tested: summing the two results (Naive (Sum)) or taking the max value for each priority level (Naive (Max)). When comparing the two, it is possible to observe that there is a slight improvement, approximately 1% in the global metrics when summing the results of the prioritization. There is, however, a concern when looking at the confusion matrices Figure 6b,c. The misclassified high priority and mid-level priority class cases are mostly being labeled as normal, with these errors being more frequent in the sum-based approach. Considering both results (global metrics and detailed results per priority classes), we decided to pursue the study using the Naive (Sum) approach. Nonetheless, in contrast with the baseline priority, it is possible to see the importance of including a diagnostic branch for the correct assessment of the lesion priority and how its quality affects the results. In fact, this implicit form of domain knowledge is essential in clinical practice, where the clinician first attributes a diagnostic category (even if in broader terms) and then assigns a priority level for referral. Comparing these results with the upper bound obtained when using the diagnostic ground-truth labels (Figure 6a), they show a difference of under 3% for accuracy and weighted F1 metrics but close to 9% for macro F1 score. Observing the confusion matrix, this is mainly due to the misclassification of HP examples (22 correctly classified samples using GT vs. 16 and 14 for KM Max and Sum, respectively), suggesting that there is still margin for improving the higher priority class results. This is further confirmed when comparing the metrics for the high priority and mid priority classes in Table 12. Here, in the normal class, the metrics are almost on par with the Naive (GT); however, in the high priority class, there is a great drop in the performance. Furthermore, it is also possible to observe that, when it comes to the mid priority class, the Naive (GT) values are lower than the Naive (Sum) and Naive (Max). This signifies that this approach is suitable for the classification of high priority and normal classes but is to a less extent for the mid priority class.

Regarding the Simple Approach, several alpha and beta values were tested, having the best results been found for alpha equal to 2 and beta to 1. When comparing these results with the best Naive approach (Sum), it is possible to affirm that the concern with the high priority and mid priority classes is still unresolved and that the performance for the normal priority worsened. Although the baseline priority model achieves higher sensitivity in priority classes and the diagnostic curriculum with summing domain knowledge (Naive Sum) achieves good F1-score in normal class, the Simple Approach that combines both information does not obtain better results, against our expectations. This suggests that the inclusion of a direct prioritization route actually hinders the overall performance, indicating that features learned in this branch are not so priority-discriminant as the ones that account for the diagnostic categories, especially considering the inclusion of explicit domain knowledge. Interestingly, the last approach tested (Combined), despite not obtaining better global metrics, achieves the best results for the mid-priority class and the predictions on high priority are more acceptable than the previous model (Simple), since the misclassifications of high priority are on the priority class instead of normal. However, this comes at the cost of having worse results for the normal class.

**Figure 7:**
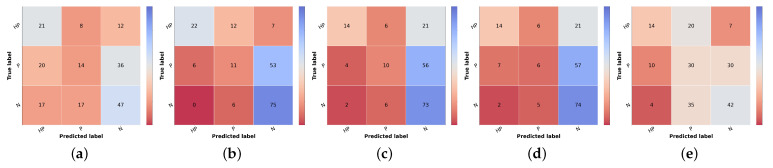
Confusion matrices of test set 2 for the different risk prioritization approaches tested. (**a**) Baseline Priority. (**b**) Naive GT. (**c**) Naive (Sum). (**d**) Simple Approach. (**e**) Combined Approach.

To the best of our knowledge, there are no reports of the prioritization task of skin lesions, so the final prioritization results were further confirmed in test set 2, as shown in Table 13, Table 14 and Figure 7. We recall that this is a subset of test set 1, where three different dermatology experts validated samples regarding their priority level and the overall class distribution is closer to balanced. Due to these facts, the more directly comparable metric is the macro F1 score since accuracy and weighted F1 are more sensitive to the underlying class distributions. Thus, comparing both test sets regarding macro F1, the first noteworthy result is that, although most results are slightly worse for test set 2, the baseline priority and the Combined Approach are higher in this set, 41% vs. 32% and 44% vs. 36%, respectively. However, this might be because these approaches have lower sensibility and precision when it comes to the normal class. Since the dataset is more balanced, an incorrect prediction in this class has less weight in the metrics compared with test set 1. Regarding the Naive (Sum) Approach, even though it has lower metrics than in test set 1, the same behavior is observed in Naive GT values, which represents the maximum possible performance in this approach.

Regarding the approaches where differential diagnosis and knowledge map are included, we see a drop in macro F1 (and, expectedly, in accuracy and weighted F1), which may be indeed a more reliable result for real-world performance, considering the validation of its labels. This comparison highlights the importance of building a sound validation strategy for any learning model, especially when dealing with a highly variable target such as dermatological case prioritization. Different hospitals and clinicians assign different levels according to available resources, previous experience, and other factors. In this context, data curation is critical, even if relevant experts cannot validate all samples. Nonetheless, data-centric methods and the inclusion of domain knowledge (either implicitly or explicitly) may help to circumvent these limitations, as shown here, improving the priority baseline by incorporating diagnostic classification (itself improved by using automatically cropped images and curriculum learning) and exploring direct relationships between differential diagnosis and expected priority distributions in the form of a knowledge map provided by experts.

## 5. Conclusions and Future Work

This paper proposes different strategies for the relevant and yet-unexplored task of automatic dermatological case prioritization based on skin lesion images and metadata. Retrospective data from the Portuguese National Health System was used, consisting of dermatological images and metadata (age and sex) from 13 different differential diagnostic classes and three priority levels (normal, priority, and high priority) attributed in the referral process for dermatology specialty consultation in public hospitals. Besides the natural diversity from different acquisition settings, the dataset includes the variability regarding the prioritization levels, which are dependent on the hospital resources to book the consultations, besides the individual clinician’s experience.

This work proposes an innovative framework for prioritization, leveraging different forms of implicit and explicit domain knowledge. The range from preprocessing operations and methodologies for training a Deep Neural Network (namely an EfficientNetB3 fine-tuned after ImageNet pretraining, as suggested in the study of Nedelcu et al. [13]). These include different learning schemes to obtain the differential diagnosis score and baseline priority score. As a particular contribution, we studied the inclusion of a novel knowledge map, provided by the dermatology experts, to improve the final priority level prediction.

Considering the real-world dataset used and the diversity of dermatological images obtained, the first experiment concerned the preprocessing operations needed in terms of lesion segmentation to improve the diagnosis classification. In this study, the best results were obtained when using cropped images with 30% tolerance (accuracy 38.77%, weighted F1 41.40%, and macro F1 27.50%, for test set 1), when compared with the original images and with cropped images with 0%, 10%, and 50% tolerance.

In terms of learning schemes for differential diagnosis classification, the cumulative strategy of curriculum learning provided the best results, achieving 48.75% accuracy, and 49.64% and 33.55% weighted and macro F1 for test set 1, respectively, when compared with flat the classification approach and both hierarchical approaches (two and three levels). Moreover, the inclusion of patients’ metadata in the differential diagnosis classification models was also studied, showing that it was not beneficial for the experiments tested, against our expectations.

Regarding the prioritization branch experiments, the priority baseline was established using the images cropped with 30% tolerance and no metadata, similar to what was found to return the best results for the differential diagnosis branch (test set 2—accuracy 42.71%, weighted F1 40.96%, and macro F1 40.51%). This baseline was highly surpassed when considering only the diagnostic branch with the knowledge map (Naive Sum), reaching in test set 2 an accuracy of 50.52%, weighted F1 of 44.39%, and macro F1 of 43.61%. Against our expectations, the Simple Approach global results, which incorporate both diagnostics with the knowledge map, and prioritization models, although superior to the baseline (accuracy of 48.96%, weighted F1 of 41.17%, and macro F1 of 40.35%), did not improve in comparison with Naive (Sum). Nevertheless, the Combined Approach achieved the best general results regarding weighted (44.93%) and macro F1 (43.93%) and the priority class achieved better sensitivity results. However, this result comes at the cost of having worse results for the normal class. From the clinical point of view, it might be preferable to choose the Combined Approach, as it gives more importance to the results for priority classes.

In what concerns future work, we identify different open research lines, namely regarding data acquisition. The DermAI project was able to design a new mobile acquisition software to support even non-specialists in capturing higher quality and more standardized lesion images. We believe that a new iteration of the proposed framework using newly acquired data in real-world settings will overcome some of the limitations shown by the current dataset, especially regarding image focus and variability. Moreover, following the potential demonstrated by the experiments with image cropping based on automatic lesion segmentation, we believe there is still margin for improvement in this important task, resorting to specific image processing techniques that could not be explored in this work. Regarding the learning schemes, although curriculum learning returned the overall best results, the shared clinical features of some diagnostic categories lead us to believe that hierarchical classification deserves to be further explored. In this context, we think an iterative approach where we use the misclassifications at a specific iteration to group fine-grained categories into higher levels could yield interesting results. Finally, concerning the inclusion of explicit domain knowledge, the standard deviation information should be also considered in the model.

In conclusion, we firmly believe that existing pre-trained models, especially for image classification, encompass more-than-enough complexity for most tasks, even the most complex ones such as dermatological case prioritization. On the other hand, research should focus on data-centric approaches to extract the most value from existing data or to improve the acquisition process and on strategies to use essential domain knowledge, be it to inspire new learning schemes or to include it explicitly to guide the predictions.

## Figures and Tables

**Table 1:** DermAI train and test dataset distribution considering the differential diagnosis and priority level (N—Normal; P—Priority; HP—High Priority; Tot—Total).

Class	Train Dataset				Test1 Dataset				Test2 Dataset			
	N	P	HP	Tot	N	P	HP	Tot	N	P	HP	Tot
1 SebKer	893	54	2	949	226	8	3	237	12	8	3	23
2 ActKer	350	56	10	416	89	14	0	103	10	14	0	24
3 Nev	444	46	5	495	114	8	0	122	11	8	0	19
4 MolCont	54	2	0	56	7	8	0	15	2	8	0	10
5 Haem	41	13	2	56	7	6	1	14	6	6	1	13
6 UncNeop	162	32	2	196	37	4	9	50	5	4	9	18
7 Dermfib	103	9	0	112	22	5	1	28	5	5	1	11
8 SLent	38	1	0	39	7	2	0	9	7	2	0	9
9 PenFib	77	14	0	91	21	1	1	23	11	1	1	13
10 VWart	137	17	0	154	36	1	1	38	11	1	1	13
11 OtMlNp	47	21	25	93	3	6	14	23	0	6	14	20
12 BCC	8	37	0	45	1	7	3	11	1	7	3	11
13 MM	13	9	22	44	0	0	8	8	0	0	8	8
Total	2367	311	68	2746	570	70	41	681	81	70	41	192

**Table 2:** Knowledge map distribution per differential diagnosis.

Class	Differential Diagnosis	Mean (%)			Std (%)		
		N	P	HP	N	P	HP
1 SebKer	Seborrheic Keratosis	80	17	3	0	7.7	7.7
2 ActKer	Actinic Keratosis	57	30	13	19	12	21
3 Nev	Nevus, Non-neoplastic	67	30	3	19	23	8
4 MolCont	Molluscum Contagiosum	60	40	0	57	45	12
5 Haem	Haemangioma	67	23	10	31	21	12
6 UncNeop	Neoplasm Unc. Behavior	40	50	10	39	28	12
7 Drmfib	Dermatofibroma	84	13	3	17	12	8
8 SLent	Solar Lentigo	84	13	3	17	12	8
9 PenFib	Pendulum Fibroma	93	7	0	15	15	0
10 VWart	Viral Warts	87	10	3	12	12	8
11 OtMalNeop	Other Malignant Neoplasm	9	42	49	17	23	21
12 BCC	Basal Cell Carcinoma	26	57	17	24	22	8
13 MM	Malignant Melanoma	0	10	90	0	12	12

**Table 3:** Metrics score of test set 1 for segmentation effect in lesion classification (in %).

Experiment	Accuracy	Weighted F1	Macro F1
Original images	37.59	40.49	26.11
Cropped with no tolerance	34.80	37.63	26.66
Cropped with 10% tolerance	33.77	36.63	25.02
Cropped with 30% tolerance	**38.77**	**41.40**	27.50
Cropped with 50% tolerance	37.59	41.36	**28.56**

**Table 4:**
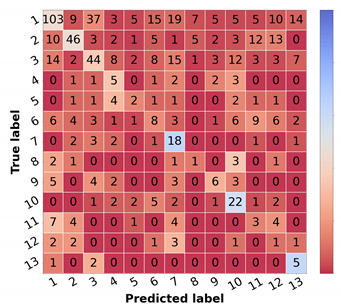
Resulting metrics and confusion matrix of test set 1 for a differential diagnosis model trained on cropped images with 30% tolerance (in %).

Classes	Sensitivity	Precision	F1-Score
1 SebKer	43.46	68.67	53.23
2 ActKer	44.66	63.89	52.57
3 Nev	36.07	44.44	39.82
4 MolCont	33.33	17.24	22.73
5 Haem	14.29	14.29	14.29
6 UncNeop	16.00	17.78	16.84
7 Drmfib	64.29	25.00	36.00
8 SLent	11.11	7.14	8.70
9 PenFib	26.09	30.00	27.91
10 VWart	57.89	36.67	44.90
11 OtMalNeop	13.03	8.57	10.34
12 BCC	9.09	2.44	3.85
13 MM	62.50	16.67	26.32

**Table 5:** Metrics score of test set 1 for differential diagnosis approaches using cropped images with 30% tolerance (in %).

Experiment	Accuracy	Weighted F1	Macro F1
Nedelcu et al. [13]	42.71	44.04	28.65
Flat	38.77	41.40	27.50
B-CNN (Two-level)	40.23	42.53	30.52
B-CNN (Three-level)	41.70	43.45	29.95
Curriculum Learning	**48.75**	**49.64**	**33.55**

**Table 6:** Resulting metrics of test set 1 for the differential diagnosis model trained with curriculum learning (in %), considering images (img), or images and metadata (img + meta) as the input.

Classes	Sensitivity		Precision		F1-Score	
	Img	Img + Meta	Img	Img + Meta	Img	Img + Meta
1 SebKer	**59.49**	54.01	68.12	**69.95**	**63.51**	60.95
2 ActKer	62.14	**71.84**	**62.14**	53.62	**62.14**	61.41
3 Nev	**47.54**	43.44	**54.21**	47.75	**50.66**	45.49
4 MolCont	**40.00**	26.67	31.58	**33.33**	**35.29**	29.63
5 Haem	7.14	**14.29**	25.00	**50.00**	11.11	**22.22**
6 UncNeop	14.00	**22.00**	16.67	**16.92**	15.22	**19.13**
7 Drmfib	**57.14**	50.00	**32.65**	31.11	**41.56**	38.36
8 SLent	11.11	11.11	4.00	**10.00**	5.88	**10.53**
9 PenFib	17.39	17.39	**30.77**	28.57	**22.22**	21.62
10 VWart	**57.89**	55.26	**52.38**	38.89	**55.00**	45.65
11 OtMalNeop	**26.09**	13.04	**13.95**	11.11	**18.18**	12.00
12 BCC	18.18	**36.36**	13.33	**30.77**	15.38	**33.33**
13 MM	**50.00**	25.00	33.33	**40.00**	**40.00**	30.77

**Table 7:** Metrics score of test set 1 for the differential diagnosis learning schemes with metadata (in %).

Experiment	Accuracy	Weighted F1	Macro F1
Flat + Metadata	36.56	39.66	26.07
B-CNN (Two-level) + Metadata	39.65	41.36	27.91
B-CNN (Three-level) + Metadata	42.00	44.22	30.90
Curriculum Learning + Metadata	**47.14**	**47.46**	**33.16**

**Table 8:** Results concerning some related skin lesion diagnosis studies.

Study	Dataset	Modalities	Classes	Acc.	Sens.	Spec.	Weight-F1	Macro-F1	AUC
Esteva et al. [4]	Several	CI, DI	9 ${}^{a}$	55.40	-	-	-	-	-
Menegola et al. [14]	EDRA ${}^{1}$, ISIC 2016 ${}^{2}$	DI	3 ${}^{b}$	-	-	-	-	-	84.50
Yap et al. [28]	Private	MI, DI, Md	5 ${}^{c}$	72.00	-	-	-	-	-
Kawahara et al. [5]	EDRA ${}^{1}$	CI, DI, Md	5 ${}^{d}$	-	60.40	91.00	-	-	89.60
Fisher et al. [39]	Dermofit ${}^{3}$	CI	10 ^*e*^	87.10	-	-	-	-	-
Barata et al. [40]	ISIC 2017 ${}^{2}$	DI	3 ${}^{f}$	-	-	-	-	-	87.40
Nedelcu et al. [23]	EDRA ${}^{1}$, ISIC 2019 ${}^{2}$, Dermofit ${}^{3}$	MI, DI, Md	5 ^*e*^	79.20	63.80	92.60	-	-	-
Nedelcu et al. [13]	DermAI	MI, AI	13	42.71	-	-	44.04	28.65	-
Curriculum Learning (proposed)	DermAI	MI, AI	13	48.75	48.75	91.10	49.64	33.55	-

^1^ EDRA 7-Point Criteria Evaluation, ^2^ ISBI Challenge/ISIC Skin Lesion Analysis towards Melanoma Detection, ^3^ Edinburgh Dermofit. Modalities: MI—macroscopic images, CI—clinical images, DI—dermoscopic images, AI—anatomical images, Md—metadata. ^*a*^ Cutaneous lymphoma; benign dermal tumors, cysts, and sinuses; malignant dermal tumor; benign epidermal tumors, hamartomas, milia, and growth; malignant and premalignant epidermal tumors; genodermatoses and supernumerary growths; inflammatory conditions; benign melanocytic lesion; malignant melanoma. ^*b*^ Benign, basal cell carcinoma, and melanoma. ^*c*^ Nevus, melanoma, basal cell carcinoma, squamous cell carcinoma, pigmented benign keratoses. ^*d*^ Basal cell carcinoma, nevus, melanoma, miscellaneous, seborrheic keratoses. ^*e*^ Actinic keratosis, basal cell carcinoma, squamous cell carcinoma, intraepithelial carcinoma, melanoma, melanocytic nevus/mole, seborrheic keratosis, pyogenic granuloma, hemangioma, and dermatofibroma. ^*f*^ Seborrheic keratosis, nevi, and melanoma.

**Table 9:** Metrics score of test set 1 for prioritization approaches (in %).

Experiment	Accuracy	Weighted F1	Macro F1
Original images	43.17	51.41	29.70
Cropped with 30% tolerance	**48.02**	**56.06**	**31.66**
Original images + Metadata	20.55	26.25	17.76
Cropped with 30% tolerance + Metadata	27.02	35.58	20.45

**Table 10:**
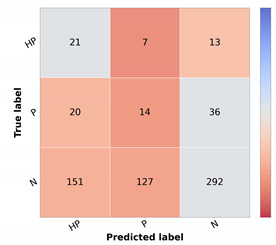
Resulting metrics and confusion matrix of test set 1 for baseline priority model (in %).

Classes	Sensitivity	Precision	F1-Score
HP	51.22	10.94	18.03
P	20.00	9.46	12.84
N	51.23	85.63	64.11

**Table 11:** Global metrics score of test set 1 for final priority level prediction (in %).

Experiment	Accuracy	Weighted F1	Macro F1
Baseline Priority	48.02	56.06	31.66
Naive GT	82.53	81.81	56.40
Naive (Max)	78.41	77.83	47.07
Naive (Sum)	80.18	78.74	47.86
Simple Approach	77.24	76.39	43.46
Combined Approach	48.31	56.77	35.60

**Table 12:** Resulting metrics (%) of test set 1 of each final priority class for the different risk prioritization approaches.

Models	Sensitivity			Precision			F1-Score		
	HP	P	N	HP	P	N	HP	P	N
1 Baseline Priority	51.22	20.00	51.23	10.94	9.46	85.63	18.03	12.84	64.11
2 Naive GT	53.66	15.71	92.81	70.97	18.03	89.81	61.11	16.79	91.29
3 Naive (Max)	39.02	15.71	88.95	31.37	21.57	87.56	34.78	18.18	88.25
4 Naive (Sum)	34.15	14.29	91.58	41.18	20.83	87.15	37.33	16.95	89.31
5 Simple Approach	34.15	8.57	88.77	31.82	11.32	86.64	32.94	9.76	87.69
6 Combined Approach	34.15	42.86	50.00	21.54	10.20	88.51	26.42	16.48	63.90

**Table 13:** Global metrics score of test set 2 for final priority level prediction (in %).

Experiment	Accuracy	Weighted F1	Macro F1
Baseline Priority	42.71	40.96	40.51
Naive GT	56.25	51.02	51.81
Naive (Sum)	50.52	44.39	43.61
Simple Approach	48.96	41.17	40.35
Combined Approach	44.79	44.93	43.93

**Table 14:** Resulting metrics (%) of test set 2 of each final priority class for the different approaches.

Models	Sensitivity			Precision			F1-Score		
	HP	P	N	HP	P	N	HP	P	N
1 Baseline Priority	51.22	20.00	58.02	36.21	35.90	49.47	42.42	25.69	53.41
2 Naive GT	53.66	15.71	92.59	78.57	37.93	55.56	63.77	22.22	69.44
3 Naive (Sum)	34.15	14.29	90.12	70.00	45.45	48.67	45.90	21.74	63.20
4 Simple Approach	34.15	8.57	91.36	60.87	35.29	48.68	43.75	13.79	63.52
5 Combined Approach	34.15	42.86	51.85	50.00	35.29	53.16	40.58	38.71	52.50

## Data Availability

The DermAI dataset is not publicly accessible due to confidentiality and privacy reasons.
